# Impact of patient position on the outcomes of percutaneous neprolithotomy for complex kidney stones

**DOI:** 10.1590/S1677-5538.IBJU.2018.0163

**Published:** 2018

**Authors:** Fabio Carvalho Vicentini, Rodrigo Perrella, Vinicius M. G. Souza, Marcelo Hisano, Claudio Bovolenta Murta, Joaquim Francisco de Almeida Claro

**Affiliations:** 1 Departamento de Urologia, Setor de Endourologia e Litíase, Hospital Brigadeiro, São Paulo, SP, Brasil

**Keywords:** Kidney Calculi, Nephrolithotomy, Percutaneous, Supine Position, Prone Position

## Abstract

**Purpose:**

To evaluate the impact of the patient position on the outcomes of PCNL among patients with complex renal stones.

**Material and Methods:**

From July 2011 to July 2014, we collected prospective data of consecutive patients who underwent PCNL. We included all patients with complex stones (Guy's Stone Score 3 or 4 (GSS) based on a CT scan) and divided them based on the position used during PCNL (prone or supine). The variables analyzed were gender, age, body mass index, ASA score, stone diameter, GSS, number of punctures, calyx puncture site, intercostal access and patient positioning. Complications were graded according to the modified-Clavien Classification. Success was considered if fragments ≤ 4mm were observed on the first postoperative day CT scan.

**Results:**

We analyzed 240 (46.4%) of 517 PCNL performed during the study period that were classified as GGS 3-4. Regarding patient positions, 21.2% were prone and 79.8% were supine. Both groups were comparable, although intercostal access was more common in prone cases (25.5% vs 10.5%; p=0.01). The success rates, complications, blood transfusions and surgical times were similar for both groups; however, there were significantly more visceral injuries (10.3% vs 2.6%; p=0.046) and sepsis (7.8% vs 2.1%; p=0.042) in prone cases.

**Conclusion:**

Supine or prone position were equally suitable for PCNL with complex stones and did not impact the success rates. However, supine position was associated with fewer sepsis cases and visceral injuries.

## INTRODUCTION

Percutaneous nephrolithotomy (PCNL) is the gold standard treatment for large kidney stones (1, 2), taking the place of morbid and invasive open surgery. Much discussion has emerged in the recent literature about patient positioning. Since Valdivia-Uria study in 1987 (3), the use of a supine position was recognized as feasible, and approximately 10 years later, good outcomes were reported by the same group (4), making this approach more accessible and even more popular with recent variations (5). The possibility of treating the patient without changing the position to prone is most widely accepted as its best virtue (6).

Prone PCNL with upper pole access has been accepted as the standard for treating complex stones, and the applicability of supine PCNL for these cases is still controversial (7). There are only a few randomized studies that compared both positions, and they showed few differences in outcomes and complications (8, 9). Considering the low number of staghorn calculi included in these studies, this question cannot be answered by the current literature.

The aim of this study was to evaluate the impact of the patient position on the outcomes, including complications and success ratios, of PCNL with complex stones, using Guy's stone score classification (10).

## MATERIALS AND METHODS

We reviewed retrospectively our prospective data collected for all patients who underwent PCNL in our institution from July 2011 to July 2014. Informed consent was obtained from patients before the surgery, and the study protocol was approved by the institutional review board.

For this study, we included all patients with complex stones, defined as grade 3 or 4 of the Guy's Stone Score (GSS) (10, 11), based on the preoperative CT scan analysis. Guy's 3 (GSS3) was defined as multiple stones in a patient with abnormal collector system anatomy, or a stone in a calyceal diverticulum, or partial staghorn calculus (stone evolving the renal pelvis plus at least two but not all calices). Guy's 4 (GSS4) was defined as a complete staghorn calculus (all calices and the pelvis occupied by stones) or any stone in a patient with spina bifida or a spinal injury leading to a neurogenic bladder. The determination of the GSS grade was made by two urologists during the preoperative consultation and again just before surgery by a senior urologist and a resident, according to the preoperative CT findings. In the case of disagreement, the senior urologist's opinion was the final one. GSS 3 and 4 were considered complex stones based on previous results showing that these groups have more complications and inferior outcomes than GSS 1 and 2 (11).

Positioning was chosen according to the surgeon's preference. Our institution is a large volume center with 7 dedicated endourologists and 3 urology residents per year. All surgeons are trained and experienced with PCNL in both decubitus. The positions that were used for PCNL were the classic prone position (12), the complete supine (csPCNL) position (13), and the classic Valdivia (3) or the Galdakao modified-Valdivia (4). The last three positions were distinguished as the supine group.

The variables analyzed were gender, age, body mass index (BMI), ASA score, stone diameter, Guy's Stone Score (GSS), number of punctures, calyx puncture site, supracostal access, operative time and patient positioning.

### Preparation and Operative Technique

All patients had urine cultures collected at least 30 days before the procedure. All of the non-colonized began prophylactic oral antibiotics (nitrofurantoin 100 mg bid) 7 days before surgery and preoperatively received third-generation cephalosporin during the induction of anesthesia, and the patients with positive cultures received therapeutic antibiotics, according to the results, 7 days before and in the induction.

Despite the position, the surgical technique was similar for all cases. Procedures were carried out under general anesthesia, beginning with a cystoscopy and placement of a 6 Fr ureteral catheter in the ipsilateral ureter, then a retrograde pyelography and subsequent renal puncture under fluoroscopy guidance. Briefly, for the prone position, the patient was initially in a lithotomy position for cystoscopy and ipsilateral ureteral catheter placement. Then, a Foley catheter was inserted and the patient was turned prone with bolsters under the arms and the pelvis. For complete supine positioning, patients were positioned in supine position with the posterior axillary line located just outside the border of the surgical table, and the flank was extended to increase the space between the last rib and the iliac crest. The csPCNL procedures were performed without a rolled towel under the flank, and the patients remained in the same position during the entire procedure without a lithotomy position, even for cystoscopy. For classic Valdivia positioning, a bolster was used under the ipsilateral flank, without using lithotomy positioning, while for the Galdakao modified Valdivia, patients were in a lithotomy position.

Calyx choice was done after retrograde pyelography and CT scan analysis. A puncture was performed with an 18 gauge needle and a 0.038 hydrophilic straight guidewire was used. Ultrasound was used to evaluate the puncture window when any abdominal organ was in a more lateral position. Tract dilation was performed with fascial dilators (double shot technique, numbers 10, 20 and 30 Fr, sequentially), and a 30 Fr Amplatz® sheath was placed. Nephroscopy was performed with a 26 Fr rigid nephroscope (Olympus, Japan), and stone fragmentation and suction were performed with an ultrasonic lithotripter (CyberWand Dual Ultrasonic Lithotriptor®, Gyrus, Olympus, Japan). An intraoperative stone-free status was verified with fluoroscopy. A 16 Fr nephrostomy tube was placed at the end of the procedure only in cases of bleeding, residual stones, solitary kidney, suspected pelvic injury, or multiple tracts. A 6 Fr ureteral catheter was routinely left in place; in cases of ureteropelvic junction with significant edema, extensive pelvic injury, or ureteral manipulation, a 4.8 Fr x 26 cm ureteral stent was used instead. Ropivacaine 1% 20 mL was injected on the tract for pain control.

### Outcome Evaluation

Operative time was considered as the beginning of the cystoscopy for ureteral catheter insertion to the end of the nephrostomy placement or sheath removal in the case of a tubeless procedure. Blood transfusion was considered for patients with signs of hypovolemia, refractory to crystalloid reposition, peri or postoperatively. Fever was defined as an axillary temperature > 37.8°C. Complications were graded according to the modified Clavien Classification, and Clavien scores ≥ 3 were considered as major complications (14). A non-contrast CT scan and routine serum exams were performed during the first postoperative day (POD1) in all cases. The success rate was defined as the absence of residual symptomatic fragments > 4 mm in the CT of the first postoperative day (POD1). Stone-free status was defined as no residual fragment at the CT scan.

#### Statistical analysis

Statistical analysis was performed using SPSS® version 20 (SPSS, Inc., Chicago, IL, USA). The tests used in the univariate analyzes were a chi-squared test and Fisher's Exact test for categorical measures and Student's t-test for independent sample continuous parameters. The level of statistical significance was 0.05.

## RESULTS

Of the 517 PCNL performed in the study period, 241 (46.6%) with GGS 3 or 4 were included in the study. Regarding patient positions, 21.2% (51) PCNL were performed in a prone position and 79.8% (190) in a supine position ((80% (152) in complete supine position, 6.8% (13) in a classic Valdivia and 13.1% (25) in a Galdakao position)).

Both groups were similar according the mean age, BMI, gender, ASA Score, stone diameter and number of punctures. Patient's demographic characteristics are listed in Table-1. There was a higher rate of supra 12^th^ costal access in the prone group compared with the supine position (25.5% vs. 10.5% respectively, p = 0.01). The surgical time, success rates, major Clavien complications and blood transfusions were similar between the groups (Table-2). There were no colon injuries in both groups, and there were two deaths (Clavien 5) due to septic complications, one in each group. The total rate of complications was 23.7%, and all complications are listed in Table-3.

**Table 1:** Demographic and clinical characteristics of patients. Distribution according to a prone or supine position.

		Supine	Prone	P
Number of patients		190	51	
Age (years ± SD)		48.04±12.2	46.7±12.5	0.51
BMI		28.2±15.8	27.8±6.1	0.87
Male		35.1%	23.5%	0.124
Female		64.9%	76.5%	
**ASA**				
	ASA I	32.6%	31.4%	0.34
	ASA II	60%	66.6%	
	ASA III	7.4%	2.0%	
Stone Diameter (cm)		32.9±11.4	63.5±11.6	0.08
Guy's Stone Score 3		76% (144)	61% (31)	0.33
Guy's Stone Score 4		24% (46)	39% (20)	
**Number of punctures**				
	1	69.8%	76.4%	0.34
	2	23.3%	21.6%	
	3	6.9%	2%	
Supra 12^th^ puncture*		10.5%	25.5%	0.01

* Statistically significant

**Table 2:** Outcomes of the pcNL for complex stones (Guy's Stone Score 3 or 4) according to a prone or supine position.

	Supine	Prone	P
Immediate Success Rate			
(fragments ≤ 4 mm on the CT scan on POD1)	38.5%	27.7%	0.1689
Clavien ≥ 3	9.6%	9.8%	0.9545
Surgical time (mean ± SD (min))	134.1 (53.4)	138.2 (42.7)	0.5770

**Table 3:** Complications in PCNL.

Complications		Supine	Prone	Total	p
Minor complications (Pain, isolated fever, perinephric bruise, bleeding after tube removal, bronchial spasm)		8.4% (16)	3.9% (2)	7.4% (18)	0.28
Blood transfusion		9.0% (16)	15.2% (7)	9.1% (23)	0.219
Pulmonary thromboembolism		0	1.9% (1)	0.4% (1)	0.53
Parenchymal visceral injury (Liver)*		0	1.9% (1)	0.4% (1)	0.53
Hollow visceral injury*		0	0	0	0.153
Pleural injury*		3.5% (5)	9.8% (5)	4.1% (10)	0.023
	Hydrothorax	2.1% (4)	5.8% (3)	1.5% (7)	
	Pneumothorax	0.5% (1)	3.9% (2)	0.8% (3)	
	Chest tube drain	0.5% (1)	5.8% (3)	1.6% (4)	
	Expectant treatment	2.1% (4)	3.9% (2)	2.4% (6)	
Any injury (thoracic/abdominal)		2.6% (5)	10.3% (6)	4.5% (11)	0.046
Ureteral stone migration		2.6% (5)	1.9% (1)	2.4% (6)	0.786
Sepsis		2.1% (4)	7.8% (4)	3.3% (8)	0.042
Severe sepsis and death (Clavien 5)		0.5% (1)	1.9% (1)	0.8% (2)	0.318

* Mann-Whitney U test for any visceral injury supine vs. prone: p=0.046

Considering visceral injuries, there were no hollow visceral injuries, the same number of thoracic events and only one abdominal event (trans-hepatic puncture). Comparing the supine and prone groups we found a significant difference, favoring supine interventions, with lower risk of any abdominal or thoracic injuries (p = 0.046) (Table-3). When considering infectious complications, sepsis had a significantly lower relative risk in supine PCNL when compared to prone cases (p = 0.042).

## DISCUSSION

In our study, we aimed to answer the question whether the supine PCNL is as good of an option as prone PCNL for complex kidney stones. Supine PCNL has proven to be suitable for simple renal stones, offering similar outcomes but in a faster manner, as reported by a few prospective studies (8, 9). Conversely, there has been controversial data regarding the outcomes for complex stones when the prone or supine approaches were compared (15-17).

Herein, we verified that supine PCNL seems to be suitable for complex stones as well as prone PCNL, since the immediate success, complications, transfusion rates and operative times were similar between the groups. However, sepsis and visceral injuries rates were significantly higher in the prone group, showing a possible safer profile for the supine position. Our hypothesis is that during supine position, there is lower chance of pyelovenous urinary backflow due to lower intrarenal pressure compared to the prone cases, since the irrigation flow through the Amplatz® sheath is intuitive. It could explain the lower sepsis rate observed in supine cases, but the lack of postoperative culture data may have limited this conclusion. More visceral injuries observed in prone cases possibly are a consequence of a higher rate of intercostal punctures, which is expected since kidneys are more cranially arranged in the abdomen in prone decubitus (18). Sofer et al. also showed that there is great approachability to the upper calyx through the inferior calyx, reducing the need for intercostal punctures when using supine decubitus (19). Our outcomes reflect the reality of our institution, which may be different in other places; however, our findings are important for supporting not only our surgical options but also those of many groups worldwide, that are starting a supine PCNL program. Our findings improve the evidence for supine position use during PCNL for complex kidney stones.

To date, there have been only a few studies on the impact of positioning during regular PCNL of complex stones and the subsequent outcomes (15-17). Using the same CROES global study in the PCNL database, Astroza et al. (15) retrospectively analyzed the influence of the position in the outcomes of PCNL for complex stones. In this large series, surgical times were shorter (P < 001) and the stone-free rate was higher (P < 001) for patients in the prone position. There were no differences in complication rates. Gokce et al. (16), in a non-randomized prospective trial comparing prone versus supine position for staghorn stone, concluded that outcomes regarding success and complications were similar, but surgical times were significantly shorter and hemoglobin drops were significantly lower in the supine group. Interestingly, in this series, endoscopic combined intra-renal surgery (ECIRS) was used in almost 70% of the cases. The authors propose that a supine position should be considered as a primary treatment option in staghorn stone cases for PCNL since outcomes are similar but supine cases are faster and have less bleeding. Our findings are more in accordance with this last study, suggesting that supine PCNL has a safer profile than prone PCNL, similar to other institutions (20-22). However, our cases were pure complete supine, without inclusion of any case where ECIRS had been used.

Our findings are also in accordance with the most recent meta-analysis that evaluated prone or supine decubitus during PCNL. Falahatkar et al. evaluated 7733 patients in 22 studies (12 RCT) and verified that both positions had similar success rates, operation times, complication rates, urinary leaking and hospital stays (20). However, patients received fewer blood transfusions (OR: 0.72; 95% CI: 0.55 - 0.94; P = 01) and had lower fever rates (OR: 0.65; 95% CI: 0.52 - 0.80; p < = 0.001) in supine PCNL.

Our immediate success rate for PCNL in complex stones was relatively low for both groups. One possible explanation is that we controlled the CT scan on the POD 1, rigorous criteria for the evaluation of success, that certainly impact the real stone free rate. In our institution, it is cost effective to consider 4 mm as the limit for defining success, so we adopted this as the criteria for success versus stricter criteria of 2 mm. The CROES PCNL Global Study (23) showed a final success rate of 75.7%, however, in the CROES study the success rates were commonly determined by conventional radiography and after auxiliary procedures, with only 14% of stone-free patients confirmed by CT. Plain radiography has low sensitivity and many RF are missed. One of the strengths of our study is the performed CT on POD1 in 100% of the patients which more precisely reflects the real results of PCNL and allows us to effectively compare what occurred after PCNL.

As for limitations, our study was not randomized, and the groups had different numbers of patients. The lack of a significant difference in the outcomes may be the result of low statistical power (e.g., small sample size) rather than the absence of a difference. The use of effective flexible nephroscope was limited to cases with no significant bleeding, which affected the success rate. Cases where ECIRS had been used were not included. The position choice was made just before surgery by the senior urologist. The criteria for choosing prone was not clear but may eventually have been reserved for the most complex cases since supracostal upper pole access in a prone position has been the recommended access for these kinds of stones. The other indication for a prone position was for resident teaching, since the majority of cases were in the supine position. The lack of criteria for positioning may be a limitation. However, the strengths may compensate the limitations. Our study included a large number of patients, the demographic data were similar in both groups, and we used a complete prospective database that reduced the chances of bias on the similarity of the outcomes regarding positioning. Surgeries were performed by experienced endourologists, not only by one surgeon. Another positive aspect of our study was all patients had a pre and postoperative non-contrast CT scan, making the outcome evaluations more reliable.

Supine PCNL can be performed in any patient with complex kidney stones. Currently literature shows no limits, similar results and complications compared to prone position. It seems to have anesthetic advantages and should be considered in obese, children, high-risk patients with compromised cardiopulmonary status, and those with skeletal deformities.

Until a multicentric randomized study including both positions with standardized pre and postoperative evaluations is available, with the current literature, and the aid of our present study, there is good evidence that the use of supine position may be a good option for PCNL for complex kidney stones.

## CONCLUSION

The supine position does not negatively impact the outcomes of PCNL for complex kidney stones. Moreover, it may be associated with a safer profile due to fewer sepsis and organ injury cases.
